# Supplementary material for: Investigating ADR mechanisms with Explainable AI: a feasibility study with knowledge graph mining
Source: BMC Med Inform Decis Mak. 2021 May 26;21:171. doi: 10.1186/s12911-021-01518-6 (PMC8157660; doi:10.1186/s12911-021-01518-6)
Supplement: Supplementary file 1 — Additional file 1. Table S1: Positive JRip rules learned from the DILI expert classification. Table S2: Positive JRip rules learned from the SCAR expert classification. Table S3: Features associated with DILI extracted from JRip rules, and presented to experts for evaluating their explanatory potential. Table S4: Features associated with SCAR extracted from JRip rules, and presented to experts for evaluating their explanatory potential. [file 12911_2021_1518_MOESM1_ESM.pdf]

Supplementary information

Title: "Investigating ADR mechanisms with Explainable AI: A feasibility study with knowledge graph mining"

Authors: Emmanuel Bresso[†], Pierre Monnin[†], Cédric Bousquet, François Calvier, Ndeye-Coumba Ndiaye, Nadine Petitpain, Malika Smaïl-Tabbone, Adrien Coulet[*]

[†] Equal contributors
[*] Correspondance: adrien.coulet@inria.fr

This supplementary document includes:

- Table S1: Positive JRip rules learned from the DILI expert classification.
- Table S2: Positive JRip rules learned from the SCAR expert classification.
- Table S3: Features associated with DILI extracted from JRip rules, and presented to experts for evaluating their explanatory potential.
- Table S4: Features associated with SCAR extracted from JRip rules, and presented to experts for evaluating their explanatory potential.

Name	*Rule*	*Support*	*Nb of FP*
Rule1	¬($\xrightarrow{\text{involvedIn}}$ Pathway $\xrightarrow{\text{contains}}$ Compound $\xrightarrow{\text{interactsWith}}$ **Cytochrome P450 2A6**) ∧ **Cytochrome P450 3A4** ⇒ ⊕	106.79	14.81
Rule2	¬($\xrightarrow{\text{involvedIn}}$ Pathway $\xrightarrow{\text{contains}}$ Compound $\xrightarrow{\text{interactsWith}}$ **Cytochrome P450 2A6)** ∧ **Vitamin digestion and absorption** ∧<br>¬($\xrightarrow{\text{involvedIn}}$ Pathway $\xrightarrow{\text{associatedWith}}$ Disease $\xrightarrow{\text{interactsWith}}$ **Calcium signaling pathway**)∧<br>¬($\xrightarrow{\text{targets}}$ Protein $\xrightarrow{\text{cellularComponent}}$ Integral component of plasma membrane) ⇒ ⊕	24.27	4.11
Rule3	¬($\xrightarrow{\text{involvedIn}}$ Resource $\xrightarrow{\text{contains}}$ Drug $\xrightarrow{\text{contains}}$ Phenols)∧<br>¬($\xrightarrow{\text{binds}}$ Resource $\xrightarrow{\text{associatedWith}}$ Drug $\xrightarrow{\text{associatedWith}}$ Resource) ∧<br>¬($\xrightarrow{\text{associatedWith}}$ Resource $\xrightarrow{\text{associatedWith}}$ Resource) ⇒ ⊕	37.98	11.52
Rule4	$\xrightarrow{\text{interactsWith}}$ Enzyme $\xrightarrow{\text{produces}}$ Drug $\xrightarrow{\text{involvedIn}}$ **Biosynthesis of secondary metabolites** ∧<br>$\xrightarrow{\text{interactsWith}}$ Enzyme $\xrightarrow{\text{cellularComponent}}$ Endoplasmic reticulum ⇒ ⊕	14.68	0.82
Rule5	$\xrightarrow{\text{associatedWith}}$ Drug $\xrightarrow{\text{associatedWith}}$ Resource $\xrightarrow{\text{associatedWith}}$ Resource ∧<br>¬($\xrightarrow{\text{interactsWith}}$ Enzyme $\xrightarrow{\text{molecularFunction}}$ Oxidoreductase activity) ⇒ ⊕	10.47	1.65
Rule6	$\xrightarrow{\text{targets}}$ Protein $\xrightarrow{\text{molecularFunction}}$ Oxidoreductase activity ∧<br>$\xrightarrow{\text{involvedIn}}$ Resource $\xrightarrow{\text{contains}}$ Organic amino compound $\xrightarrow{\text{interactsWith}}$ **Cytochrome P450 1A1** ⇒ ⊕	5.86	0.82

Table S1: JRip rules learned from the DILI expert classification. Only positive rules (*i.e.*, rules with ⊕ on the right-hand side) are generated. *Support* of rules is the number of examples satisfying the rule. *Nb of FP* is the number of false positives. These two metrics are float because of weights associated with examples to balance classes in the training. Note that the second and third attributes of Rule2 and the first attribute of Rule 5 are path patterns that only contain general classes. We exclude this kind of attributes of our analysis because they are not sufficiently explanatory. In bold font are entities (final or not) of path patterns and neighbors.

Name	*Rule*	*Support*	*Nb of FP*
Rule1	$\neg(\xrightarrow{\text{targets}}$ Protein $\xrightarrow{\text{molecularFunction}}$ Iron ion binding$)\ \wedge \xrightarrow{\text{interactsWith}}$ Enzyme $\xrightarrow{\text{biologicalProcess}}$ Electron transport $\wedge$ $\neg(\xrightarrow{\text{targets}}$ Protein $\xrightarrow{\text{biologicalProcess}}$ Positive regulation of cell proliferation $\Rightarrow \oplus$	88.82	8.11
Rule2	$\neg(\xrightarrow{\text{targets}}$ Protein $\xrightarrow{\text{molecularFunction}}$ Iron ion binding$)\ \wedge$ $\xrightarrow{\text{transportedBy}}$ Protein $\xrightarrow{\text{cellularComponent}}$ Membrane $\Rightarrow \oplus$	34.12	3.38
Rule3	$\xrightarrow{\text{targets}}$ Protein $\xrightarrow{\text{molecularFunction}}$ Nucleotide binding $\wedge$ **Cytochrome P450 2B6** $\neg(\xrightarrow{\text{interactsWith}}$ Enzyme $\xrightarrow{\text{molecularFunction}}$ Oxidoreductase activity$)$ $\Rightarrow \oplus$	42.70	8.11
Rule4	$\neg(\xrightarrow{\text{involvedIn}}$ Resource $\xrightarrow{\text{contains}}$ Sulfonamide $\xrightarrow{\text{transportedBy}}$ **Solute carrier organic anion transporter family member 1A2)** $\wedge$ $\neg(\xrightarrow{\text{interactsWith}}$ **Cytochrome P450 3A5)** $\wedge\ \neg$**(Calcium signaling pathway)** $\wedge$ $\neg(\xrightarrow{\text{transportedBy}}$ Protein $\xrightarrow{\text{molecularFunction}}$ ATPase activity$)$ $\Rightarrow \oplus$	52.70	19.60
Rule5	$\xrightarrow{\text{involvedIn}}$ **Calcium signaling pathway** $\wedge\ \neg(\xrightarrow{\text{involvedIn}}$ **Antifungal agents)** $\Rightarrow \oplus$	8.36	0.68

Table S2: JRip rules learned from the SCAR expert classification. Only positive rules (*i.e.*, rules with $\oplus$ on the right-hand side) are generated. *Support* of rules is the number of examples satisfying the rule. *Nb of FP* is the number of false positives. These two metrics are float because of weights associated with examples to balance classes in the training. In bold font are entities (final or not) of path patterns and neighbors.

Table S3: Features (neighbors and path patterns) associated with DILI extracted from JRip rules, and presented to experts for evaluating their explanatory potential with regard to DILI.

1:	$\neg($ $\xrightarrow{\text{involvedIn}}$ Pathway $\xrightarrow{\text{contains}}$ Compound $\xrightarrow{\text{interactsWith}}$ **Cytochrome P450 2A6**)
2:	**Cytochrome P450 3A4**
3:	**Vitamin digestion and absorption**
4:	$\neg($ $\xrightarrow{\text{involvedIn}}$ Pathway $\xrightarrow{\text{associatedWith}}$ Disease $\xrightarrow{\text{interactsWith}}$ **Calcium signaling pathway**)
5:	$\neg($ $\xrightarrow{\text{targets}}$ Protein $\xrightarrow{\text{cellularComponent}}$ Integral component of plasma membrane
6:	$\neg($ $\xrightarrow{\text{involvedIn}}$ Resource $\xrightarrow{\text{contains}}$ Drug $\xrightarrow{\text{contains}}$ Phenols)
7:	$\xrightarrow{\text{interactsWith}}$ Enzyme $\xrightarrow{\text{produces}}$ Drug $\xrightarrow{\text{involvedIn}}$ **Biosynthesis of secondary metabolites**
8:	$\xrightarrow{\text{interactsWith}}$ Enzyme $\xrightarrow{\text{cellularComponent}}$ Endoplasmic reticulum
9:	$\neg($ $\xrightarrow{\text{interactsWith}}$ Enzyme $\xrightarrow{\text{molecularFunction}}$ Oxidoreductase activity)
10	$\neg($ $\xrightarrow{\text{targets}}$ Protein $\xrightarrow{\text{molecularFunction}}$ Oxidoreductase activity
11:	$\xrightarrow{\text{involvedIn}}$ Resource $\xrightarrow{\text{contains}}$ Organic amino compound $\xrightarrow{\text{interactsWith}}$ **Cytochrome P450 1A1**

Table S4: Features (neighbors and path patterns) associated with SCAR extracted from JRip rules, and presented to experts for evaluating their explanatory potential with regard to SCAR.

1:	$\neg($ $\xrightarrow{\text{targets}}$ Protein $\xrightarrow{\text{molecularFunction}}$ Iron ion binding)
2:	$\xrightarrow{\text{interactsWith}}$ Enzyme $\xrightarrow{\text{biologicalProcess}}$ Electron transport
3:	$\neg($ $\xrightarrow{\text{targets}}$ Protein $\xrightarrow{\text{biologicalProcess}}$ Positive regulation of cell proliferation
4:	$\xrightarrow{\text{transportedBy}}$ Protein $\xrightarrow{\text{cellularComponent}}$ Membrane
5:	$\xrightarrow{\text{targets}}$ Protein $\xrightarrow{\text{molecularFunction}}$ Nucleotide binding
6:	**Cytochrome P450 2B6**
7:	$\neg($ $\xrightarrow{\text{interactsWith}}$ Enzyme $\xrightarrow{\text{molecularFunction}}$ Oxidoreductase activity)
8:	$\neg($ $\xrightarrow{\text{involvedIn}}$ Resource $\xrightarrow{\text{contains}}$ Sulfonamide $\xrightarrow{\text{transportedBy}}$ **Solute carrier organic anion transporter family member 1A2**)
9:	$\neg($ $\xrightarrow{\text{interactsWith}}$ **Cytochrome P450 3A5**)
10	$\neg($**Calcium signaling pathway**)
11:	$\neg($ $\xrightarrow{\text{transportedBy}}$ Protein $\xrightarrow{\text{molecularFunction}}$ ATPase activity)
12:	$\xrightarrow{\text{involvedIn}}$ **Calcium signaling pathway**
13:	$\neg($ $\xrightarrow{\text{involvedIn}}$ **Antifungal agents**)
